# Genetic Variants in *CD36* Involved in Fat Taste Perception: Association with Anthropometric and Clinical Parameters in Overweight and Obese Subjects Affected by Type 2 Diabetes or Dysglycemia—A Pilot Study

**DOI:** 10.3390/nu15214656

**Published:** 2023-11-03

**Authors:** Marica Franzago, Paola Borrelli, Marta Di Nicola, Liborio Stuppia, Ester Vitacolonna

**Affiliations:** 1 Department of Medicine and Aging, School of Medicine and Health Sciences, “G. D’Annunzio” University, Via dei Vestini, Chieti-Pescara, 66100 Chieti, Italy; marica.franzago@unich.it; 2 Center for Advanced Studies and Technology (CAST), “G. D’Annunzio” University, Chieti-Pescara, 66100 Chieti, Italy; stuppia@unich.it; 3 Laboratory of Biostatistics, Department of Medical, Oral and Biotechnological Sciences, “G. D’Annunzio” University, Chieti-Pescara, 66100 Chieti, Italy; paola.borrelli@unich.it (P.B.); marta.dinicola@unich.it (M.D.N.); 4 Department of Psychological, Health and Territorial Sciences, School of Medicine and Health Sciences, “G. D’Annunzio” University, Chieti-Pescara, 66100 Chieti, Italy

**Keywords:** *CD36*, *CLOCK*, *BMAL1*, rs1761667, rs1984112, gene–diet interaction, nutrition, type 2 diabetes, obesity, nutrigenetics

## Abstract

Obesity and overweight represent a growing health problem worldwide. Genes regulating the intake and metabolism of different nutrients can positively or negatively influence the efficacy of nutritional interventions against obesity and its complications. The aim of this study was to assess changes in anthropometric and clinical parameters and the adherence to a Mediterranean diet (MedDiet) over time in relation to nutrigenetic variants in overweight or obese subjects affected by Type 2 Diabetes (T2D) or dysglycemia, who were included in a nutritional program. A total of 23 subjects were included in this study. Clinical parameters, physical activity levels, and the adherence to a MedDiet were evaluated at baseline, at 6 (T6), and at 12 months (T12) during and after a diet/lifestyle intervention. In a single blood sample from each subject, rs1984112 (*A*>*G*) and rs1761667 (*G*>*A*) in *CD36*; rs7950226 (*G*>*A*) in *BMAL1*; and rs1801260 (*A*>G), rs4864548 (*A*>*G*), and rs3736544 (*G*>*A*) in *CLOCK* were genotyped with Real-Time PCR. Significant associations were observed between *CD36* rs1761667 and weight (*p* = 0.025), hip circumference (*p* = 0.042), triglycerides (*p* = 0.047), and HbA1c (*p* = 0.012) at baseline. Moreover, the genotype *AA* in *CD36* rs1761667 was significantly associated with a lower BMI when compared to *G* carriers at baseline, at T6, and also at T12. In addition, subjects with the *AA* genotype at *CD36* rs1984112 had significantly lower levels of HbA1c (*p* = 0.027) than the *GG* and *AG* genotypes at baseline. These results show that variants in *CD36* can have an impact on anthropometric and clinical parameters in overweight or obese subjects affected by T2D or dysglycemia, and that it might influence the success of the diet/lifestyle intervention.

## 1. Introduction

Overweight and obesity, which are chronic, progressive, and relapsing conditions associated with an elevated risk of non-communicable diseases (NCDs, such as cardiovascular disease, type 2 diabetes (T2D), hypertension, and metabolic syndrome), have become a major global public health challenge [1,2]. Environmental and social factors, as well as the genetic susceptibility represented by the individual’s genotype, can influence an individual’s predisposition to the development and maintenance of obesity [1].

The increased global burden of obesity requires specific strategies to prevent weight gain, to induce weight loss, and to improve obesity comorbidities. Unfortunately, successful weight loss with behavioral and nutritional interventions, as well as long-term weight maintenance, are difficult to achieve. In this context, the effects of several single nucleotide polymorphisms (SNPs) located near or within genes regulating food intake, lipid metabolism, glucose homeostasis, and insulin signaling on metabolic improvement, weight gain/loss, and insulin resistance have been demonstrated [3,4]. Interestingly, disturbances in the circadian gene network can lead to the onset and development of obesity and some accompanying comorbidities [5]. In fact, SNPs located in circadian-related genes such as Circadian Locomotor Output Cycles Kaput (*CLOCK*) and Brain and Muscle Aryl Hydrocarbon Receptor Nuclear Translocator-Like Protein-1 (*BMAL1*), which interacts with both dietary intake and obesogenic behavior, can affect metabolic health [6,7,8], modifying body weight regulation [9,10]. In this regard, from a nutrigenetics point of view, minor allele *C* carriers of rs1801260 in the *CLOCK* gene have lower body weight loss than *TT* carriers [11].

In addition, it is known that obese individuals can display fat chemosensory dysfunction, and that the SNPs correlated with fat chemosensation are located in the *CD36* gene [12].

The *CD36* gene, which codes for an integral membrane glycoprotein identified as a taste receptor for fat [13], is intimately involved in several processes related to fatty acid and lipid metabolism-sensing in the organism. Genetic variants of this gene can contribute to an increased risk of obesity by modifying an individual’s food preference and intake [14,15,16,17]. Among these, rs1761667, which is characterized by a substitution of allele *G* for *A*, has been associated with a decrease in sensitivity to fatty acids [15,18,19,20], a decrease in the expression of CD36 protein, and a decrease in metabolism [21,22,23]. A recent study showed that adherence to a healthy dietary pattern (a diet with high fiber, fish, and dairy products) can affect cardiometabolic risk factors and MetS risk in the *A*-allele carrier in rs1761667 [24].

Thus, gene–diet interactions seem to play an important role in the treatment of obesity, but are typically only partially assessed nowadays. Thus, the aim of this study is to evaluate the effects of a nutritional and lifestyle intervention based on nutrigenetic variants in candidate genes (namely, *CD36* rs1984112 *A*>*G*, *CD36* rs1761667 *G*>*A*, *BMAL1* rs7950226 *G*>*A*, *CLOCK* rs1801260 *A*>*G*, *CLOCK* rs4864548 *A*>*G*, and *CLOCK* rs3736544 *G*>*A*) on the anthropometric and clinical parameters of 23 overweight or obese subjects affected by T2D or impaired glucose regulation (IGR) over a one-year period. Variants located in the genes involved in clock systems (*CLOCK* and *BMAL1*) were selected, considering that circadian disruptions may contribute to different metabolic-related traits and that polymorphisms in the *CD36* gene that are related to lipid detection may be associated with interindividual variability in body weight regulation. To the best of our knowledge, this is the first study on overweight or obese subjects affected by T2D or IGR that is based on this nutrigenetic panel, evaluating not only the success of body weight loss but also the feasibility of a personalized nutritional approach.

## 2. Materials and Methods

### 2.1. Study Design and Participants

A total of 23 overweight or obese individuals affected by T2D or IGR were recruited at the Diabetes, Nutrition, and Metabolism Unit at the “Gabriele d’Annunzio” University Hospital in Chieti, Italy. This study received the approval of the Ethics Committee of the Province di Chieti and Pescara, in accordance with the Helsinki Declaration. Before undertaking the protocol, all objectives and modalities were clarified to the participants, and written informed consent was therefore obtained.

The inclusion criteria were overweight or obese (BMI ≥ 25 and ≥ 30 kg/m^2^, respectively) subjects (male and female, adults: age ≥ 18) affected by T2D or Impaired Glucose Regulation (IGR; Impaired Fasting Glucose or Impaired Glucose Tolerance).

The exclusion criteria were as follows: subjects suffering from Type 1 Diabetes, Eating Behavior Disorders, impaired renal and hepatic function, or other conditions that might have interfered with the development and completion of the protocol.

### 2.2. Educational and Nutritional Intervention

At baseline (T0), all subjects participated in an educational and nutritional program, in which each individual was actively involved in face-to-face individual and group-based interventions to promote a healthy diet and lifestyle.

In detail, the first visit consisted of a clinical check-up and the collection of the individual’s medical history, which included data on demographic characteristics. In addition, each participant was instructed to complete a self-monitoring diary [25,26,27,28]. The diaries were reviewed by a physician during the next meeting. In addition to the face-to-face individual intervention, educational group sessions with small groups of up to 10 people were conducted in the presence of the physician. The group-based intervention was crucial to involve the participants in improving their lifestyle. This educational and nutritional program included three main goals to achieve, as previously described [29]: (i) improve the composition of meals, prioritising vegetables as well as whole foods and limiting highly processed foods; (ii) weight loss of at least 7–10%; (iii) plan at least 150 minutes a week of moderate intensity physical activity [25,26,27,28,30].

### 2.3. Anthropometric and Clinical Measurements

Data on anthropometric (body weight and height, BMI, waist and hip circumferences) and clinical parameters (including fasting glucose, HbA1c (glycated hemoglobin A1c), lipid profile, and blood pressure) were collected at baseline (T0), at 6 months (T6) and at 12 months (T12) after the nutritional education program.

### 2.4. Questionnaires

At baseline, circadian typology (Chronotype)—defined as the interindividual circadian attitude—was assessed using a 5-item version of the Morningness–Eveningness Questionnaire (MEQr). According to Italian cut-off criteria, the MEQr generates three categories of chronotypes, usually divided by the terms: (i) “morning” (19–25 score), (ii) “evening” (4–10 score), and (iii) “intermediate” types (11–18 score) [31]. The MEQr characterizes subjects based on individual differences in wake/sleep cycle patterns and the time of the day that people feel or perform best.

Adherence to the Mediterranean diet (MedDiet) was assessed using a validated 14-item questionnaire (PREDIMED) that provides a range of possible scores, specifically: no adherence (score ≤ 5), medium adherence (6 ≤ score ≤ 9), and maximum adherence (score ≥ 10) [32]. Moreover, levels of physical activity were evaluated using a short version of the International Physical Activity Questionnaire (IPAQ), which showed three different intensity levels, namely: low, moderate, and high PA [33]. Physical activity and MedDiet adherence were evaluated at baseline, T6, and T12.

### 2.5. Gene and SNP Selection

Nutrigenetic variants from three genes, identified in previous studies as associated with T2D, obesity, lipid metabolism, and dietary intake were selected. Specifically, two of these variants in the *CD36* gene, namely rs1984112 (*A*>*G*) and rs1761667 (*G*>*A*), were involved in fat intake regulation [34,35]. Moreover, rs7950226 (G>A) in *BMAL1*, rs1801260 (*A*>*G*), rs4864548 (*A*>*G*), and rs3736544 (*G*>*A*) in *CLOCK* were involved in obesity, CVD, MetS, sleep reduction, alterations in eating behaviors, and evening preference [9,36,37,38,39,40,41,42,43].

#### Genetic Analysis

Blood from each participant was collected for genotype analyses in a tube containing EDTA and was stored at +4 °C before analysis.

The genetic analysis was conducted at the Laboratory of Molecular Genetics, School of Medicine and Health Sciences, ‘‘G. d’Annunzio” University of Chieti-Pescara. Genomic DNA was extracted from peripheral blood lymphocytes using a MagPurix 12sAutomatedNucleicAcid Purification System (Zinexts Life Science Corp., New Taipei City, Taiwan). Nucleic acids were quantified using the Qubit assay kit on a Qubit 4 Fluorometer (Invitrogen, Thermo Fisher, Waltham, MA, USA).

To genotype the polymorphisms, molecular analyses were performed using a TaqMan SNP Genotyping Assay according to the manufacturer’s instructions (ThermoFisher Scientific, Foster City, CA, USA). The details of TaqMan SNP Genotyping assays are available in Table 1.

In detail, approximately 20 ng of DNA, 0.125 µL of TaqMan 40× concentration assay, and 2.5 µL of TaqMan Mastermix were used to amplify DNA sequences of interest on the QuantStudio 5 Real-Time PCR System (Applied Biosystem, Foster City, CA, USA). PCR conditions were 95 °C for 10 min, 40 cycles of 95 °C for 15 s, and 60 °C for 1 min.

### 2.6. Statistical Analysis

Descriptive analysis was carried out using the median and interquartile range (IQR) for quantitative variables, while frequencies and percentages were used to describe the qualitative variables. Normality distribution was tested using the Shapiro–Wilk test. To evaluate the relationships between qualitative variables, a Pearson chi square test and/or Fisher’s test was assessed. However, for quantitative variables, the Friedman test was used to determine the differences between the medians of the three time periods (Baseline, T6, and T12) and the Wilcoxon rank-sum (Mann–Whitney) test or Kruskal–Wallis test was performed to assess the differences between nutritional parameters at baseline and in genotypes (for additive, dominant, and recessive models). For significant trends, this analysis was followed by the Sign test or Dunn’s test for comparisons between median pairs for the identification of significant differences. The Bonferroni’s correction for multiple comparisons tests was applied. Several linear mixed models were used to determine the differences between male and female patients, between time periods (baseline, T6, and T12), and their interactions with the anthropometric and clinical data. Several linear mixed models were also used to determine the differences between different genotypes, between different time periods (Baseline, T6, and T12), and their interaction with the following variables: weight, BMI, and PREDIMED.

A statistical significance was set at the level of ≤0.05, unless adjustment for multiple comparisons was needed. All analyses were performed using Stata software v17 (StataCorp, College Station, TX, USA).

## 3. Results

The Demographic and clinical characteristics of the cohort of participants at baseline, at T6, and at T12 of the nutritional intervention are reported in Table 2 and Table 3, respectively.

A total of 23 subjects (n = 23, 11 males and 12 females) were included in the study. The median age of the participants was 65.0 (IQR 57.0–66.0) years. Regarding the distribution of chronotypes, 21.7% were a “morning-type” and 78.3% were an “intermediate-type”.

Our results showed significant variations over time for weight (*p* = 0.002) and BMI (*p* = 0.002; Table 3). In addition, the median PREDIMED score was 7.0 and 8.5 at baseline and at the end of the study, respectively (*p* = 0.002). Supplementary Table S1 shows the anthropometric and clinical data for females and males, separately, at baseline, T6, and T12.

Females had a significantly lower hip circumference (*p* = 0.034) and higher PREDIMED scores (*p* = 0.041) over time when compared to males (Supplementary Table S1).

Supplementary Figures S1–S3 summarize the distribution of genotypes based on additive, dominant, and recessive inheritance genetic models. The distribution of the tested SNP allele frequencies in the participant cohort compared to that in the European and general population reported to 1000 Genomes (dbSNP Short Genetic Variations) are shown in Supplementary Table S2.

Regarding *CD36* rs1761667 *(G>A)*, the additive genetic model (*AA* vs. *AG* vs. *GG*) showed that subjects carrying the *AA* genotype had a significantly lower hip circumference (*AA* 102.5 (IQR 101.0–104.0) vs. *AG* 123.0 (IQR 112.5–128.0) vs. *GG* 109.0 (IQR 104.0–114.0), *p* = 0.042; in particular, *AA* vs. *AG*, *p* = 0.028), weight (*AA* 82.8 (IQR 78.0–83.0) vs. *AG* 102.0 (IQR 92.8–114.3) vs. *GG* 89.5 (IQR 66.0–92.5), *p* = 0.025; in particular, *AA* vs. *AG*, *p* = 0.027), BMI (*AA* 28.1 (IQR 27.3–28.7) vs. *AG* 37.2 (IQR 32.4–39.9) vs. *GG* 31.3 (IQR 28.2–35.9), *p* = 0.012; in particular, *AA* vs. *AG p* = 0.006), and HbA1c (*AA* 5.8 (IQR 5.6–6.3) vs. *AG* 6.5 (IQR 5.9–7.4) vs. *GG* 7.6 (IQR 7.5–8.4), *p* = 0.012; in particular, *AA* vs. *GG*, *p* = 0.004) when compared to the *GG* and *AG* carriers at baseline.

Furthermore, using a recessive inheritance model ((*GA*+*GG*) vs. (*AA*)), the subjects carrying the *G* allele in *CD36* rs1761667 had a higher BMI (35.9 (IQR 31.3–38.0) vs. 28.1 (IQR 27.3–28.7), *p* = 0.009, respectively), HbA1c (7.2 (IQR 6.3–7.7) vs. 5.8 (IQR 5.6–6.3), *p* = 0.013, respectively) and hip circumference (114.0 (IQR 110.0–126.0) vs. 102.5 (IQR 101.0–104.0), *p* = 0.045, respectively) than *AA* carriers at baseline.

In addition, the dominant inheritance model ((*GA*+*AA*) vs. (*GG*)) of *CD36* rs1761667 demonstrated that carriers of the *A*-allele reported significantly lower levels of triglycerides (119.5 (IQR 90.0–189.5) vs. 253.5 (IQR 174.0–380.5), *p* = 0.047), HbA1c (6.3 (IQR 5.9–6.6) vs. 7.6 (IQR 7.5–8.4), *p* = 0.013) when compared to *GG* carriers at baseline.

However, regarding *CD36* rs1984112 (*A*>*G*), subjects with the *AA* genotype had significantly lower levels of HbA1c (*AA* 5.8 (IQR 5.6–6.3) vs. *AG* 6.6 (IQR 6.3–7.5) vs. *GG* 8.3 (IQR 7.4–9.2), *p*= 0.028; in particular, *AA* vs. *GG*, *p* = 0.027) than *GG* and *AG* genotypes at baseline.

Moreover, mixed linear models for additive and recessive inheritance models of *CD36* rs1761667 showed a significant relationship between genotype and BMI, as well as between the latter and the time trend (Table 4 and Table 5). In particular, homozygous subjects for the *A* allele had a lower BMI compared to *G* carriers at baseline, at T6, and at T12 (Table 4 and Table 5). We also analyzed participants’ dietary habits through four items of the PREDIMED related to fat intake (use of olive oil, sofrito, consumption of red/processed meats and butter/cream/margarine). A significant fat consumption reduction after the nutritional intervention adjusted for CD36 rs1761667 genotypes (*p* = 0.007) was found.

For the other genetic variants, a statistically significant difference was found only in the temporal trend, excluding *CLOCK* rs3736544 in the recessive inheritance model (Table 4 and Table 5).

No significant differences were detected related to SNPs in the *CLOCK* and *BMAL1* genes. Regarding questionnaires relating all the tested genetic variants, no differences were detected between PREDIMED, MEQr, or IPAQ scores assuming dominant, recessive, or additive genetic inheritance models. In addition, the interaction terms between genetic variants and time were also not significant.

## 4. Discussion

The main aim of this pilot study was to evaluate the effects of the interactions between some genetic variants and nutritional intervention on mid-term changes in anthropometric and clinical parameters in overweight or obese subjects affected by T2D or IGR over a one-year period.

The additive genetic model showed that *GG* and *AG* carriers at *CD36* rs1761667 presented with higher significant parameters such as BMI, hip circumference, and weight compared to subjects for homozygous the *A* allele at baseline.

CD36 is involved in several processes including oro-sensory perception of dietary lipids, inflammatory responses, angiogenesis, metabolism, and regulation of the metabolic pathways of insulin-resistance [44]. CD36 is a widely expressed glycoprotein that acts as a receptor for a several ligands, including saturated, mono-unsaturated, and poly-unsaturated fatty acids in taste bud cells, as well as being a transporter of long-chain fatty acids into adipose and muscle tissues [14,45]. In the past, the decreased expression of CD36 in the circumvallate taste buds of high-fat diet-induced obese rats has been related to diminished fatty taste sensitivity; consequently, the intake of dietary fat increases as a compensatory response [46]. Lower CD36 expression, induced by a currently unknown mechanism in the *AA* and *AG* genotypes at rs1761667, has been associated with food choices, lipid profiles, and adiposity parameters, as well as in reducing the release of peptide YY from taste bud cells [46]. These relationships may play a key role in the preference for energy-rich diets, obesity risk, and associated complications. The genotypic variation at rs1761667 has been emphasized as a reason for the disparity in fat perception between individuals [47]. On the other hand, several studies in different ethnic populations have shown ambivalent results regarding the association between rs1761667 and T2D, total fat consumption and fat taste perception, obesity, and metabolic syndrome [35,48,49,50,51].

It should be noted that there are differences in the frequency of minor alleles in different ethnic groups; in fact, *A* is the minor allele in Africans and Asians, while *G* is the minor allele in Caucasians and Americans. This may in part explain the differences in the genotype–phenotype relationship reported across studies, as rs1761667 may have ethnic-specific effects on the perception of fat-containing foods [20].

Interestingly*,* Pioltine et al. [52] reported that the rs1761667 in *CD36* is not related to obesity, although the *A* allele is associated with decreased fat and carbohydrate (CHO) intake in obese children and adolescents. The association of *CD36* rs1761667 with BMI and hypertension has also been studied in a cohort of adults, showing a correlation between the *AA* genotype and lower BMI as compared to *AG* and *GG* [53]. In addition, it has been shown that the *AA* genotype has a significantly lower degree of dyslipidemia, systolic blood pressure, and WC compared to *G* carriers [51]. Boghdady et al. [49] also demonstrated that the *AG* genotype is associated with coronary artery disease, raised BMI, metabolic syndrome, and T2D.

Furthermore, it has been shown that the *CD36* SNP is linked to cardiovascular risk factors, suggesting an important role in LDL-C and HDL-C metabolism [20,54,55,56].

In our study, *CD36* with the *AA* genotype at rs1761667 showed lower levels of TG when compared to *GG* and *AG*. This finding conflicts with the study of Karthi et al. [47], but it is consistent with a recent systematic review and meta-analysis [57]. Therefore, it is necessary to clarify the effects of taste perception and lipid transport on TG levels in subjects with *CD36* rs1761667. Nevertheless, further studies are required to understand if the modulation in TG levels observed in the *CD36* variant occurs at the level of taste perception or metabolism.

The present study demonstrated that homozygous subjects with the *A* allele at both *CD36* rs1761667 and rs1984112 had significantly lower HbA1c concentrations than those with the *GG* or *AG* genotypes at baseline. This observation corroborates the findings of previous studies [19,58], in which it has been shown that the *GG* genotype is significantly associated with higher plasma HbA1c as compared to the *AA* genotype of *CD36* rs1761667. Considering the role of CD36 in some aspects of fatty acid and lipid metabolism, it has been suggested that variants in this gene can influence energy homeostasis [53].

Considering the significant role of CD36 as a receptor defining the preference for fat, taste dysfunction may be responsible for abnormalities in food intake (leading to obesity) and may not be reversed by weight loss (predisposing to a relapse) [13].

In this view, this pilot study provides valuable insight into the role of CD36, showing that the *AA* genotype at rs1761667 has lower adiposity parameters such as BMI, hip circumference, and weight. Our results suggest that *CD36* variants may promote a protective metabolic profile, probably reducing protein expression.

This field of taste research provides a promising path to understand how genetic variants in fat taste preference contribute to eating habits as well as to health and disease. Although the specificity and mechanism of this receptor’s function must still be better clarified, it is plausible to suggest that knowledge of the *CD36* variants of an individual—which preferentially favor the intake of some nutrients and adversely affect the consumption of others—may help to prevent chronic diseases and also improve the personalization of interventions against obesity and obesity-related complications. This study has limitations—in particular, the sample size. The evidence of interactions between genetic variants and the nutritional intervention estimated may be unduly small (i.e., a false negative result), and may preclude the opportunity to further examine its latent efficacy.

However, this is a pilot study, which is a small-scale version of a subsequent investigation designed to test various parameters, including the feasibility of the study protocol, as well as measures and procedures [59,60]. In this view, the capabilities of the study design, the procedures, and strategies could be taken into consideration in a larger study.

Finally, future investigations with a large population (including obese subjects without T2D as a control group) and in different ethnic groups must be conducted to confirm these findings, and to shed light on the functional role of CD36 in obesity and on the success of diet/lifestyle interventions.

## Figures and Tables

**Table 1:** The TaqMan SNP Genotyping Assays.

Genetic Variants	Location	Assay
*CD36* rs1984112 (*A*>*G*)	Chr.7: 80613604 on GRCh38	C__12093946_10
*CD36* rs1761667 (*G*>*A*)	Chr.7: 80615623 on GRCh38	C___8314999_10
*CLOCK* rs1801260 (*A*>*G*)	Chr.4: 55435202 on GRCh38	C___8746719_20
*CLOCK* rs4864548 (*A*>*G*)	Chr.4: 55547636 on GRCh38	C__11821276_10
*BMAL1* rs7950226 (*G*>*A*)	Chr.11: 13296592 on GRCh38	C__11578388_10
*CLOCK* rs3736544 (*G*>*A*)	Chr.4: 55443825 on GRCh38	C__22273263_10

**Table 2:** Sociodemographic characteristics of participants.

Variable	Baseline
Age (yr)	65.0 (57.0–66.0)
Gender	
- Female	12 (52.2%)
- Male	11 (47.8%)
Employment	
- Employed	22 (95.7%)
- Unemployed	1 (4.3%)

n (%) or median and interquartile range (IQR) are shown when appropriate.

**Table 3:** Anthropometric and clinical data of participants at baseline, T6, and T12.

Variable	Baseline	T6	T12	*p*-Value ^a^
Weight (Kg)	93.0 (78.0–103.0)	92.0 (79.0–101.0) *	91.5 (79.5–104.0) *	**0.002**
BMI (kg/m^2^)	31.8 (28.1–37.8)	33.7 (27.9–37.2) *	30.4 (26.1–36.1) *	**0.002**
Waist circumference (cm)	108.5 (100.0–118.0)	107.0 (97.0–113.0)	109.0 (93.0–116.0)	0.152
Hip circumference (cm)	113.0 (104.0–126.0)	114.5 (101.5–123.0)	112.0 (99.0–120.0)	0.452
WHR	1.0 (0.9–1.0)	1.1 (1.0–1.1)	0.9 (0.9–1.0)	0.178
Systolic blood pressure (mmHg)	130.0 (110.0–150.0)	130.0 (120.0–140.0)	125.0 (120.0–137.5)	0.717
Diastolic blood pressure (mmHg)	80.0 (70.0–90.0)	80.0 (75.0–85.0)	80.0 (70.0–80.0)	0.494
PREDIMED	7.0 (7.0–8.0)	9.0 (9.0–10.0) *	8.5 (8.0–10.0) *	**0.002**
PREDIMED CLASS				0.301
- No adherence	5.3%	0.0%	0.0%	
- Adherence	78.9%	58.8%	72.2%	
- Max adherence	15.8%	41.2%	27.8%	
IPAQ				0.318
- Low	52.6%	29.4%	22.2%	
- Moderate	31.6%	47.1%	38.9%	
- High	15.8%	23.5%	38.9%	
Fasting blood glucose (mg/dL)	115.0 (105.0–140.0)	107.0 (102.0–108.0)	110.0 (102.0–125.0)	0.223
Hba1c	6.5 (5.9–7.5)	6.1 (6.0–6.3)	6.3 (5.5–6.7)	0.350
Total cholesterol (mg/dL)	199.0 (183.0–217.5)	208.5 (197.0–238.5)	193.0 (160.0–223.0)	0.751
HDL (mg/dL)	41.0 (37.0–55.0)	48.5 (41.0–55.0)	46.0 (41.0–51.0)	0.135
TG (mg/dL)	139.5 (93.5–214.5)	118.5 (94.5–187.5)	120.4 (96.0–160.7)	0.900
LDL (mg/dL)	120.4 (101.2–138.0)	132.7 (121.6–160.7)	123.8 (90.8–137.0)	0.913

n (%) or median and interquartile range (IQR) are shown when appropriate. BMI, body mass index; HDL-C, high-density lipoprotein cholesterol; LDL-C, low-density lipoprotein cholesterol; TG, triglycerides. ^a^ *p*-values are for Pearson’s chi-square test or Friedman test; * *p*-value < α/3 for Bonferroni multiple testing correction vs. Baseline. Statistically significant values are given in bold.

**Table 4:** Mixed linear model for additive genetic model. Relationship between the subjects’ BMI at baseline with those at T6 and T12 and its association with SNPs.

				*p*-Value		
*CD36* rs1984112 *A>G*	AA	AG	GG	Genotype ^a^	Time ^b^	Interaction ^c^
BMI						
Baseline	28.6 (27.6–29.4)	36.2 (31.9–38.0)	29.7 (28.2–31.3)	0.124	**0.019**	0.964
T6	27.8 (27.3–29.1)	36.0 (31.6–38.0)	29.3 (28.0–30.6)			
T12	27.9 (26.1–28.2)	35.1 (30.0–36.9)	29.1 (27.8–30.4)			
*CD36* rs1761667 *G>A*	AA	AG	GG			
BMI						
Baseline	28.0 (27.3–28.7)	37.2 (32.4–39.9)	31.3 (28.2–35.9)	**0.011**	**0.001**	0.968
T6	27.6 (26.5–28.4)	36.2 (32.7–39.3)	30.6 (28.0–36.0)			
T12	26.1 (25.4–27.9)	35.5 (31.9–38.3)	29.1 (25.5–32.3)			
*BMAL1* rs7950226 *G>A*	AA	AG	GG			
BMI						
Baseline	34.7 (26.9–44.2)	36.5 (28.5–37.9)	30.5 (28.0–35.7)	0.567	**<0.001**	0.167
T6	40.3 (28.0–45.9)	35.9 (27.3–37.2)	30.6 (27.8–36.0)			
T12	32.3 (26.7–41.6)	35.5 (29.8–36.1)	30.0 (26.1–34.6)			
*CLOCK* rs1801260 *A>G*	AA	AG	GG			
BMI						
Baseline	35.9 (29.4–37.9)	29.1 (26.7–32.4)	41.1 (27.6–41.3)	0.081	**<0.001**	0.214
T6	36.0 (30.6–37.2)	28.9 (25.0–32.7)	40.3 (27.3–40.8)			
T12	34.6 (28.2–36.0)	26.7 (24.7–30.9)	38.6 (36.9–40.4)			
*CLOCK* rs4864548 *G>A*	AA	AG	GG			
BMI						
Baseline	37.9 (37.9–37.9)	32.1 (28.7–35.9)	30.7 (26.4–38.8)	0.753	**0.008**	0.593
T6	36.5 (36.5–36.5)	33.8 (29.1–36.0)	31.6 (27.3–40.3)			
T12	36.0 (36.0–36.0)	29.3 (27.0–34.9)	30.9 (25.6–36.9)			
*CLOCK* rs3736544 *G>A*	AA	AG	GG			
BMI						
Baseline	36.6 (31.9–42.0)	29.7 (28.7–35.6)	35.4 (28.4–39.9)	0.132	**0.003**	0.387
T6	37.0 (31.9–42.0)	30.6 (29.1–35.1)	35.1 (27.7–39.3)			
T12	36.1 (27.9–46.5)	29.1 (26.1–31.9)	36.4 (27.8–38.3)			

Data are expressed as median and interquartile range (IQR). Statistically significant values are given in bold. ^a^ Groups, for each variable, the differences have been tested between Genotypes over time. ^b^ Time, for each variable, the differences have been tested between baseline, T6, and T12 of the three Genotypes. ^c^ Probability that the effects of nutritional intervention are greater in one distinct group (interaction Time × Genotype).

**Table 5:** Mixed linear model for recessive inheritance model. Relationship between the subjects’ BMI at baseline with those at T6 and T12 and its association with SNPs.

			*p*-Value		
*CD36* rs1984112 *A>G*	GG	AA+AG	Genotype ^a^	Time ^b^	Interaction ^c^
BMI					
Baseline	29.7 (28.2–31.3)	33.0 (28.6–37.9)	0.438	0.119	0.781
T6	29.3 (28.0–30.6)	35.1 (27.8–38.0)			
T12	29.1 (27.8–30.4)	31.8 (26.1–36.1)			
*CD36* rs1761667 *G>A*	AA	GG+GA			
BMI					
Baseline	28.0 (27.3–28.7)	35.9 (31.2–38.0)	**0.014**	**0.001**	0.831
T6	27.6 (26.4–28.4)	35.9 (30.6–38.0)			
T12	26.1 (25.6–27.9)	34.9 (30.0–36.9)			
*BMAL1* rs7950226 *G>A*	AA	GG+GA			
BMI					
Baseline	34.8 (26.9–44.2)	31.9 (28.5–36.7)	0.400	**<0.001**	0.877
T6	40.3 (28.0–45.9)	32.7 (27.8–36.5)			
T12	32.3 (26.7–41.6)	30.4 (26.1–36.0)			
*CLOCK* rs1801260 *A>G*	GG	AA+AG			
BMI					
Baseline	41.1 (27.6–41.3)	31.9 (28.5–36.7)	0.362	**<0.001**	0.123
T6	40.3 (27.3–40.8)	32.7 (28.0–36.5)			
T12	38.6 (36.9–40.4)	30.2 (27.0–35.5)			
*CLOCK* rs4864548 *G>A*	AA	GG+GA			
BMI					
Baseline	37.9 (37.9–37.9)	31.6 (28.2–36.7)	0.462	**0.049**	0.699
T6	36.5 (36.5–36.5)	32.7 (27.9–37.6)			
T12	36.0 (36.0–36.0)	30.2 (26.1–36.1)			
*CLOCK* rs3736544 *G>A*	AA	GG+GA			
BMI					
Baseline	36.6 (31.9–42.0)	31.2 (28.2–37.9)	0.129	0.078	0.363
T6	37.0 (31.9–42.0)	31.6 (28.0–36.5)			
T12	36.1 (27.9–46.5)	30.2 (26.0–35.6)			

Data are expressed as median and interquartile range (IQR). Statistically significant values are given in bold. ^a^ Groups, for each variable, the differences have been tested between Genotypes over time. ^b^ Time, for each variable, the differences have been tested between baseline, T6, and T12 of the two Genotypes. ^c^ Probability that the effects of nutritional intervention are greater in one distinct group (interaction Time × Genotype).

## Data Availability

The data that support the findings of this study are available from the corresponding author upon reasonable request.

## Supplementary Materials

The following supporting information can be downloaded at: https://www.mdpi.com/article/10.3390/nu15214656/s1, Table S1. Anthropometric and clinical data of females and males at baseline, at T6, and T12. Table S2. Allele frequencies in subjects compared to the general and European population. Figure S1. The distribution of genotypes in each SNP based on an additive model. Figure S2. The distribution of genotypes in each SNP based on a dominant model. Figure S3. The distribution of genotypes in each SNP based on a recessive model.
