# Supplementary material for: Genetic Variants in CD36 Involved in Fat Taste Perception: Association with Anthropometric and Clinical Parameters in Overweight and Obese Subjects Affected by Type 2 Diabetes or Dysglycemia—A Pilot Study
Source: Nutrients. 2023 Nov 3;15(21):4656. doi: 10.3390/nu15214656 (PMC10647499; doi:10.3390/nu15214656)

Figure S1. The distribution of genotypes in each SNP based on additive model.

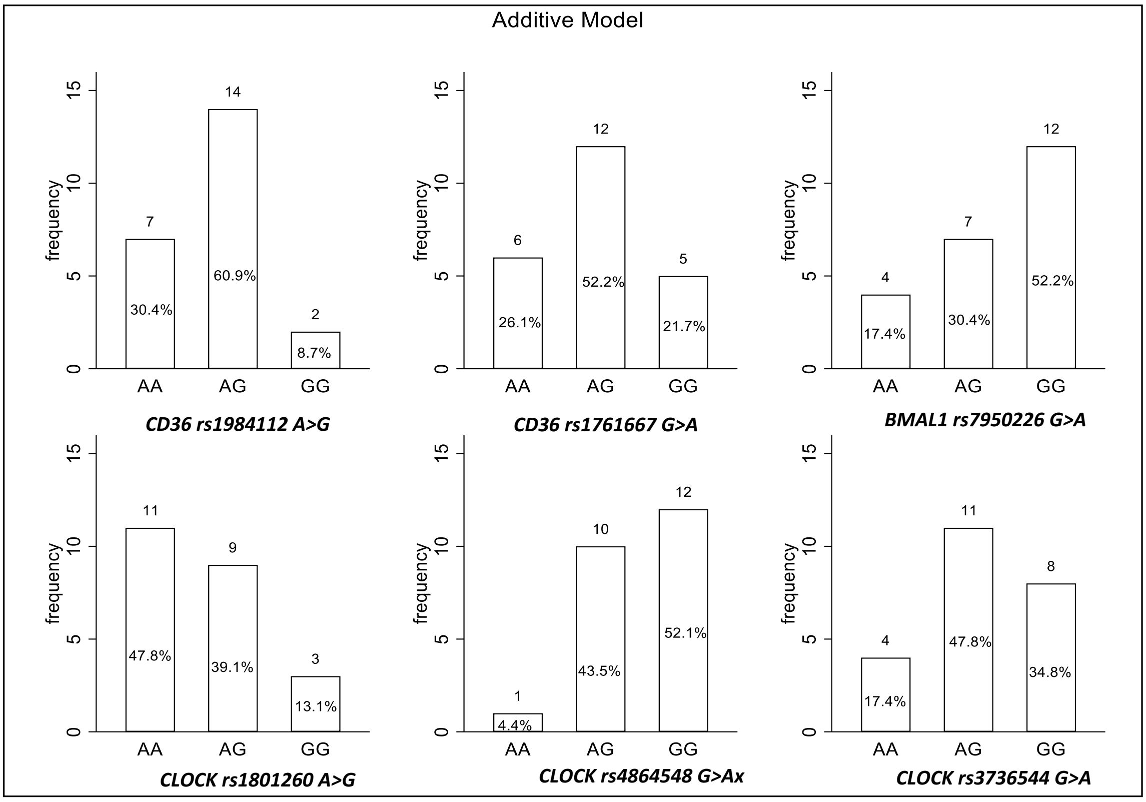

Figure S2. The distribution of genotypes in each SNP based on dominant model.

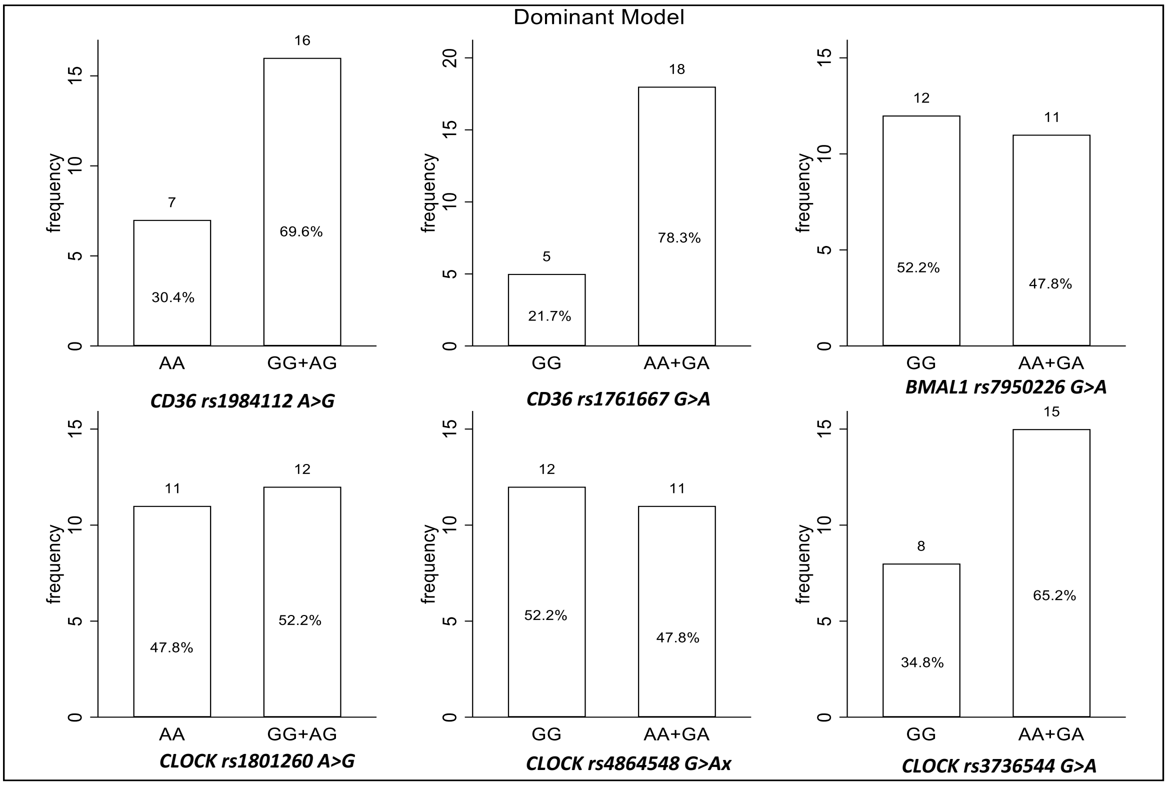

Figure S3. The distribution of genotypes in each SNP based on recessive model.

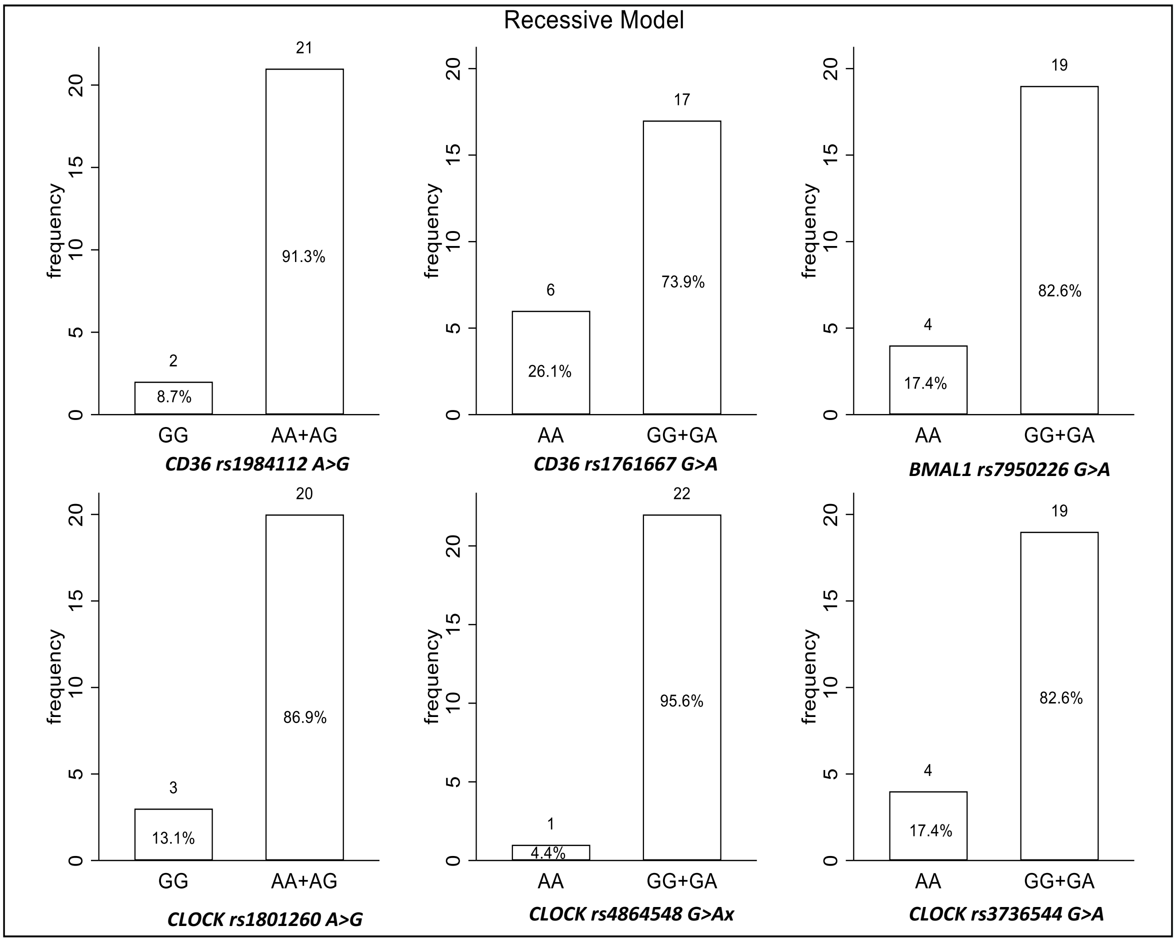

Supplement: Supplementary file 1 [file nutrients-15-04656-s001.zip › nutrients-2662010 def/supplementary figures 1-3 24.10.23.pdf]
